# Supporting Sustainable Return to Work in Large Organizations: A Cluster Randomised Feasibility Trial of a Multi-Level Intervention

**DOI:** 10.3390/ijerph23081030

**Published:** 2026-08-06

**Authors:** Fehmidah Munir, Alice Sinclair, Oliver Davis, Jeremy Dawson, Lizzie Degerdon, Jaime Delgadillo, Umesh Kadam, Joanna Yarker

**Affiliations:** 1 School of Sport, Exercise and Health Sciences, Loughborough University, Loughborough Campus, Loughborough LE11 3TU, UK; alice.sinclair@affinityhealthatwork.com; 2 Affinity Health at Work, London SW12 9NW, UK; jo.yarker@affinityhealthatwork.com; 3 Rotherham, Doncaster and South Humber NHS Foundation Trust, Doncaster DN4 8QN, UK; oliver.davis@nhs.net (O.D.); ldegerdon1@sheffield.ac.uk (L.D.); 4 Institute of Work Psychology, Sheffield University Management School, University of Sheffield, Sheffield S10 2TN, UK; j.f.dawson@sheffield.ac.uk; 5 Institute of Psychiatry, Psychology & Neuroscience, King’s College London, London WC2R 2LS, UK; jaime.delgadillo@kcl.ac.uk; 6 Public Health and Sport Sciences, Faculty of Health and Life Sciences, University of Exeter, Exeter EX4 4QJ, UK; u.kadam@exeter.ac.uk; 7 Birkbeck Business School, Birkbeck, University of London, London WC1E 7HX, UK

**Keywords:** feasibility, cluster randomised trial, sick leave, return-to-work, employer, manager, worker

## Abstract

**Highlights:**

**Public health relevance—How does this work relate to a public health issue?**

- Long-term sickness absence contributes to economic inactivity, reduced workforce participation and widening health inequalities, making it a major public health concern.
- This study addresses the need for workplace-based, multi-level interventions that support both return-to-work and sustained employment among individuals with mental and physical health conditions.

**Public health significance—Why is this work of significance to public health?**

- The study provides evidence that a theory-informed, multi-level intervention is acceptable and can be delivered in real-world organizational settings, while highlighting important recruitment and implementation challenges.
- Findings highlight the importance of evaluating sustainable work participation, alongside time-to-return outcomes in future trials.

**Public health implications—What are the key implications or messages for practitioners, policy makers and/or researchers in public health?**

- Leadership capability, manager support and organisational conditions may be important influences on successful implementation of return-to-work interventions.
- Future research and policy should prioritize scalable recruitment pathways and implementation strategies that account for organizational pressures and contextual constraints.

**Abstract:**

Long-term sickness absence is a growing public health issue, reducing workforce participation and widening health inequalities. Effective interventions must address both health and workplace factors to enable not only return-to-work but sustained employment. This study evaluated the feasibility of a multi-level workplace intervention to support sustainable RTW in large organizations. A two-arm cluster randomised feasibility trial was conducted in nine UK organizations (≥600 employees). Participants included senior leaders and HR professionals (*n* = 91), workers on long-term sick leave (*n* = 112), and line managers (*n* = 83). The IGLOo intervention combined e-learning for leaders/HR with structured toolkits for workers and managers to support RTW before and after return. Feasibility outcomes included recruitment, retention, engagement, acceptability, and usability. Secondary outcomes included RTW timing, sustained participation, mental health, and workplace factors. A mixed methods process evaluation explored implementation and context. Retention, acceptability, and usability met targets, but recruitment and engagement were lower than expected. Potential mechanisms included improved confidence, communication, and self-management. Organizational systems and pressures affected delivery. Exploratory analyses suggested different patterns of return-to-work trajectories across trial arms. Overall, the intervention was acceptable to participants who engaged with it, but recruitment barriers and organizational constraints require substantial modification before any future efficacy trial.

## 1. Introduction

Long-term sickness absence is a major and escalating public health challenge in the UK and internationally, contributing to rising economic inactivity, lost productivity, and widening health inequalities [1,2]. Mental health conditions, often co-occurring with other health conditions such as musculoskeletal problems, are leading drivers of long-term sickness absence [3,4,5], with many workers continuing to experience symptoms, reduced functioning, and work limitations after they return to work (RTW) [6,7]. These challenges create substantial societal and economic burdens [8] and highlight the need for comprehensive workplace approaches that support both RTW and long-term sustainability of work participation.

Evidence suggests that RTW interventions are most effective when symptom-focused strategies (e.g., cognitive behavioural approaches) are combined with work-directed components such as early, high-quality communication and structured planning led by line managers, regular manager contact during absence and structured support [9,10,11,12]. However, the most comprehensive RTW programmes have been developed in countries with strong legislative incentives or integrated health–social care structures (e.g., parts of Scandinavia, The Netherlands, Canada) [13,14,15], limiting transferability to the UK, where employers and line managers hold primary responsibility for absence management [16], and organizational RTW practices remain variable [17], despite national guidance from UK bodies such as NICE [18]. This gap points to the need for UK-based, evidence-informed multi-component interventions that can be implemented within diverse organizational contexts.

The IGLOo intervention was designed to address this gap through a multi-level, multicomponent approach grounded in the IGLOo model [19], which emphasizes resources and actions at the Individual, Group, Leader, Organization, and Outside-context levels (Supplementary File S1). IGLOo integrates principles from cognitive behavioural [20], communication accommodation [21], and social cognitive theories [22], and from job demands-resources concepts [23], to strengthen self-management, supportive communication, managerial practice, and job design for sustainable RTW. The intervention aims to facilitate initial RTW and to maintain participation over time, reflecting evidence that many workers face ongoing challenges for up to a year after returning [6,24].

Policy priorities in the UK increasingly emphasize early intervention, stronger employer capability, and sustained employability for people with health conditions. Government initiatives [25,26] highlight the urgent need for evidence-based practices to reduce long-term sickness absence and support people to return to and remain in work. This intervention directly responds to these priorities by offering a structured, theoretically informed approach that targets known barriers at the individual, managerial, and organizational levels.

Despite strong conceptual foundations, there have been no cluster-randomised feasibility evaluations of IGLOo within larger UK organizations to establish the practicality of delivery in large, real-world organizations, to understand implementation mechanisms and contextual barriers, or to inform a future effectiveness trial focusing on sustainability of RTW. This study therefore aimed to assess the feasibility of a cluster-randomised controlled trial of the IGLOo intervention, examining recruitment, retention, data collection, and outcome sensitivity. It also evaluated implementation in terms of acceptability, fidelity, usability, and sustainability and explored contextual influences and potential mechanisms of implementation to refine the existing IGLOo programme theory and inform the design of a future efficacy trial.

## 2. Materials and Methods

### 2.1. Study Design, Setting, and Population

We conducted a two-arm pilot cluster-randomised controlled trial with a nested mixed-methods process evaluation to assess the feasibility, acceptability, and implementation of the multi-level IGLOo RTW intervention in large UK organizations. The trial was conducted over 36 months, followed the CONSORT 2010 Extension to Pilot and Feasibility Trials [27], received Health Research Authority approval from the Leicester Central Research Ethics Committee (IRAS 313499; REC 22/EM/0143), was conducted in accordance with the Declaration of Helsinki, and was prospectively registered on 6 October 2022 (ISRCTN11788559). The trial protocol has been published [28], and a list of amendments to the protocol is available in Supplemental File S2.

A Patient and Public Involvement (PPI) group of 13 members with diverse job roles and health conditions co-designed the recruitment plan, promotional materials, and evaluation tools, helping to enhance clarity and relevance. The lead PPI contributor (LD) also joined project team meetings to ensure ongoing engagement.

#### 2.1.1. Settings, Clusters, and Randomization

Organizations were the unit of randomization used to minimize and reflect the organizational nature of the intervention. Among organizations with ≥600 employees in the Yorkshire and Humber region, England, and with at least 2% of staff on long-term sick leave in past year, 29 were eligible and recruited through NHS partnerships, sector networks, and direct outreach. Our recruitment target was ten organizations and at least four clusters per arm [29]. Ninety-four large organizations were approached, resulting in twenty-four expressions of interest. Ten organizations provided informed consent, and one withdrew prior to the start, leaving nine clusters that were randomized 1:1 to intervention or usual practice and stratified by NHS versus non-NHS status. Participants and intervention providers were unblinded due to the nature of the intervention. All participating organizations provided written consent.

#### 2.1.2. Participants and Recruitment

Participants were recruited across three phases corresponding to intervention levels. In Phase 1, HR contacts in intervention organizations only identified and invited eligible senior leaders and HR professionals via email. Those interested contacted the research team directly and subsequently provided informed consent to participate. In Phase 2, HR teams in intervention and control organizations used sickness absence records to identify workers on long-term sick leave for 15–42 days due to any mental or physical health condition, excluding those with severe mental disorders (e.g., psychosis), substance misuse, and cancer undergoing prolonged investigation, as well as workers under disciplinary procedures. Postal invitations were sent by HR to workers and parallel email invitations were sent to their line manager. Participants registered their interest electronically or by post directly to the research team and were subsequently provided with informed consent forms to register their willingness to participate. Worker–manager dyads were encouraged but not required, meaning that either party could participate independently. Workers and managers consenting in Phase 2 were followed in Phase 3 after the worker’s return.

### 2.2. Intervention

The IGLOo intervention was structured across three phases and targeted Individual, Group, Leader Organization, and Outside-context levels to support both RTW and sustained participation. Phase 1 (Organizational level) delivered a five-step online programme for senior leaders and HR professionals covering mental health and RTW foundations, integrating best practice into policy and processes, stakeholder engagement, and strategies for embedding the toolkits within existing systems.

In Phases 2 and 3 (both targeting Individual and Leader levels), workers and managers accessed mirrored online toolkits organized into six-steps: (1) managing initial sick leave, (2) maintaining communication during absence, (3) preparing to return, (4) reintegration in the first week back, (5) staying healthy at work, and (6) sustaining work participation. Each step included evidence-based guidance, action checklists, communication guides, exercises, RTW planning tools, and resources to support sustained work participation. Toolkit use was flexible to reflect stage of absence and local context. Workers were additionally offered three one-hour telephone coaching sessions delivered by trained researchers to enhance engagement with the toolkits, support goal setting and problem-solving, and address barriers to returning to work [28]. Intervention components related to Group and External Support levels were not assessed. The intervention was underpinned by cognitive behavioural theory [20], communication and accommodation theory [21], social cognitive theory [22], and job demands-resources concepts [23]. In this feasibility trial, Group and Outside-context components were not formally assessed.

### 2.3. Control Condition

Control organizations continued with usual absence and RTW procedures. Prior to randomization, organizational policies were reviewed to confirm that existing RTW processes did not duplicate or conflict with the intervention.

### 2.4. Implementation Strategies

To support study implementation, multi-level strategies were used. At organizational entry, sickness-absence and RTW policies were mapped to assess readiness and identify integration routes. As described in the intervention section, senior leaders and HR staff completed a structured online learning programme (Phase 1), which functioned as a leadership capacity-building strategy to secure buy-in and support embedding of RTW practices within organizational policies, consistent with ERIC guidance [30]. Each site nominated an internal HR lead who acted as an implementation champion, coordinating eligibility screening and invitations and serving as a liaison for implementation check-ins. Co-produced internal communications supported study awareness, and intervention organizations were encouraged to provide protected time for toolkit use, with alternative formats supplied where digital access was constrained.

Ongoing facilitation was provided by the research team through regular meetings with HR leads to monitor progress, address contextual barriers, and adapt roll-out as required. Recruitment procedures described elsewhere also functioned as an implementation strategy, enabling systematic identification and invitation of eligible workers and managers. Participant-facing conversation guides and checklists supported consistent enactment of RTW practices. Indicators of organizational integration were documented, and proposed adaptations for future scale-up were identified through the process evaluation.

### 2.5. Outcomes, Measures, and Timepoints

The primary objective was feasibility rather than effectiveness. Pre-specified stop–go “traffic light” criteria [27,31] guided progression decisions for recruitment, retention, completion, acceptability, and usability (Table 1). Feasibility outcomes comprised organizational uptake, participant recruitment, and retention and data completion at each timepoint: Phase 1 participants completed surveys at baseline, 1 month after receiving the learning, and a 6-month email follow-up regarding practice changes and use of toolkits. Phase 2 participant workers and managers completed surveys at baseline, 3, 6, 9, and 12 months. Baseline data were used to assess potential selection bias between groups. The feasibility of collecting sick-leave data via monthly text messages to participants was evaluated for completeness.

#### 2.5.1. Intervention Implementation

A nested process evaluation, informed by the Implementation Outcomes Model (IOM) [32] and Integrative Process Evaluation Framework (IPEF) [33], was conducted to assess intervention fidelity (from website analytics and participant interviews), acceptability, appropriateness, usability, sustainability, self-reported behaviour changes among workers and managers, and implementation costs. In addition, the IPEF, which incorporates realist evaluation principles, was used to guide assessment of context, mechanisms, and outcomes (CMOs). The a priori CMO configurations were specified in the published protocol [28] and programme theory [19] and informed both data collection and analysis. Data collection was designed to capture evidence relating to intervention implementation outcomes, as well as contextual factors that could influence the activation of the proposed mechanisms and outcomes across the different IGLOo levels. Data sources included website analytics, worker, manager, and leader interviews (with embedded Likert-rating questions) at phase-specific milestones (all participants invited to interview), recording of worker coaching sessions, and monthly interviews with HR contacts to document operational barriers and facilitators and contextual influences on implementation. Organizational documents and communications (e.g., emails, posters) were analysed to assess awareness-raising strategies, and the organization-wide survey captured work demands, policy awareness, mental health culture, and climate (baseline and 18 months). Qualitative interviews also focused on how organizational conditions (e.g., workload, restructuring, strikes) influenced implementation and engagement.

#### 2.5.2. Exploratory Outcomes

Exploratory RTW outcomes were defined a priori as days from the first day of the long-term sick-leave spell to the first day of partial or full RTW, and the number of days at work without a subsequent spell of long-term sick leave during the following six months, to capture both speed and sustainability. RTW status and dates were collected via surveys and monthly text messages and were triangulated by the research team. Secondary worker-reported outcomes included depression [34], anxiety [35], quality of life [36], exhaustion/burnout [37], RTW expectations [38], readiness and confidence [39], communication quality and support [40], intention to quit [41], and job satisfaction [42]. Once a worker had returned to work, questions were asked on work performance [43], readiness to stay [44], work adjustments, job crafting [45], and colleague support [46]. Secondary manager-reported outcomes included prior training and experience in sick leave/RTW management, job demands [44,47], autonomy [48,49], and confidence in managing mental health [50]. Workers and managers also completed a manager competence scale [51]. Demographic data were collected. Survey questions for leaders and HR professionals included policy awareness, job autonomy and demands, change readiness, communication, and demographics. See the protocol for a full description [28].

### 2.6. Sample Size and Analyses

As a feasibility trial, the target was to recruit 100 workers (50 per arm) from eight organizations across 18 months in order to estimate design parameters [52]. No recruitment targets were set for managers or leaders and HR professionals. Analysis focused on feasibility and exploratory evaluation. Quantitative analyses summarized feasibility indicators descriptively and explored patterns in outcomes with multilevel models accounting for organizational clustering. Time to RTW was examined using multilevel Cox regression; binary outcomes (any RTW, sustained RTW) used multilevel logistic regression; and continuous measures used multilevel linear regression with baseline adjustment where available. Sensitivity analyses included demographic covariates (age, gender, ethnicity, education) and Multiple Imputation by Chained Equations for missing data.

Interview transcripts, coaching recordings and organisational process data were analysed using template and thematic analysis. Analyses relating to feasibility and implementation outcomes were guided by the Implementation Outcomes Model (IOM). A separate realist-informed analysis was undertaken to examine context-mechanism-outcome (CMO) configurations across the Individual, Group, Leader, Organisation, and Outside levels. Qualitative data were coded against the pre-specified CMO configurations described in the published programme theory and trial protocol [28] to examine whether, how, and under what circumstances the proposed mechanisms were activated. The analysis was primarily deductive, using the published CMO configurations as an analytical framework, while inductive thematic analysis was used to identify refinements to the programme theory, additional contextual influences, and unanticipated implementation processes. The researchers OD and AS independently analysed an initial sample of transcripts, each coding interviews conducted by the other researcher. The researchers then met to compare coding and interpretations and refine the developing analytical framework. OD subsequently led the full CMO mapping across the dataset, with emerging interpretations and programme theory refinements discussed with AS and, where required, the wider research team. Any differences in interpretation were resolved through discussion and consensus. The final CMO configurations therefore represent refinement of the original programme theory based on evidence from the feasibility trial.

## 3. Results

### 3.1. Recruitment and Retention

Table 2 provides details of the nine participating organizations (fur intervention, five control). Eight were public services organizations, including three NHS Trusts. Policies on sick leave and RTW were broadly comparable at baseline, with minor procedural variations (Supplementary File S3).

**Figure 1:**
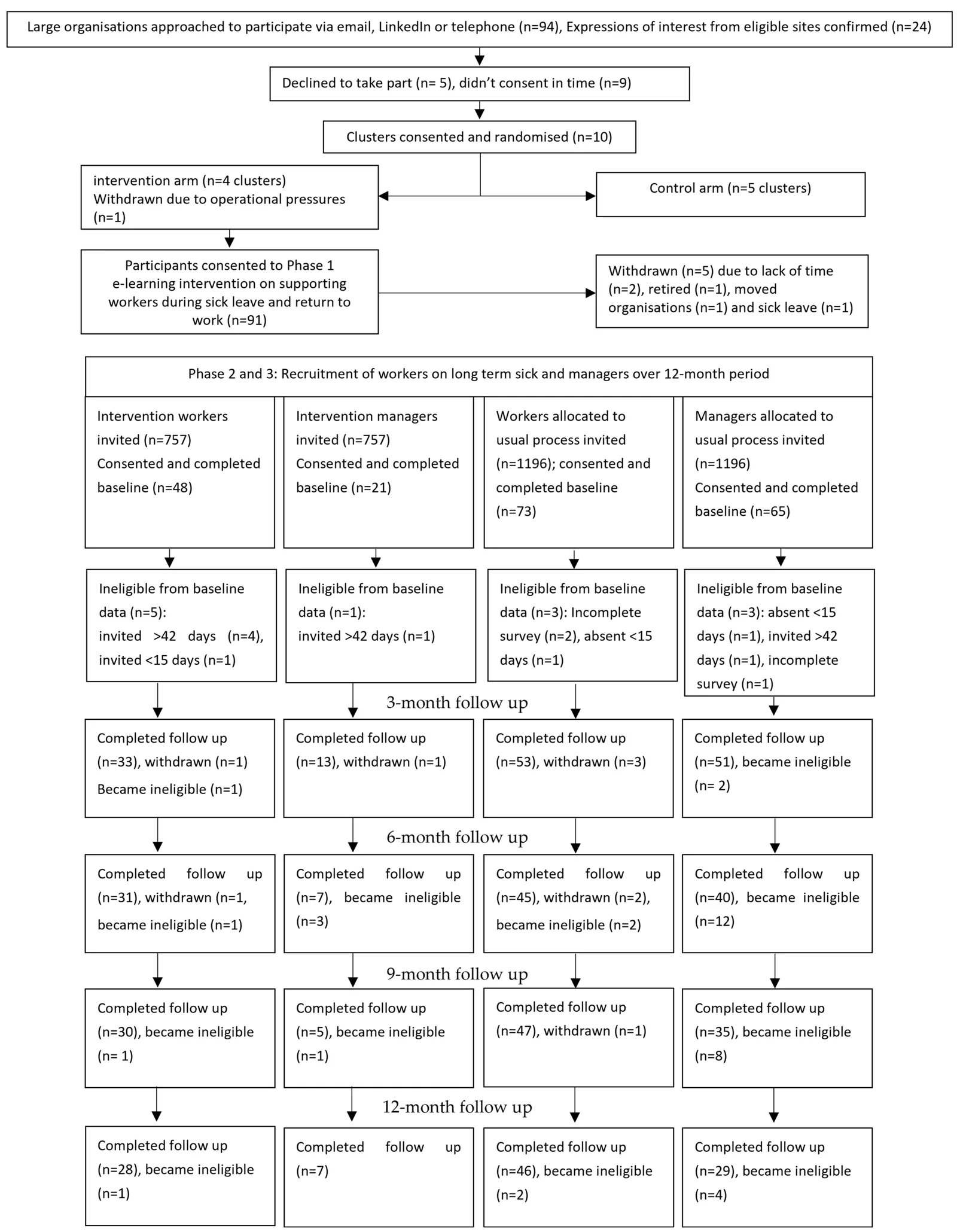
Flow diagram.

In Phase 1, 247 senior leaders and HR professionals were invited from intervention organizations; 91 (36.8%) consented and completed baseline, 41 (45.1%) completed the one-month follow-up, 23 were interviewed, and 13 provided six-month behaviour change feedback. In Phases 2–3, organizations posted 1953 invitations to eligible workers and emailed a corresponding number of managers. In total, 112 workers (43 intervention, 69 control) and 83 managers (20 intervention, 63 control) consented; no dyads were formed. Most intervention-arm participants were recruited from two organizations, and over half of the control participants were from one site (Organization 5, Table 2). Incomplete absence records prevented calculation of the proportion of all eligible workers invited. Participant flow is shown in Figure 1 and participant characteristics are given in Table 3. Across arms, the modal reason for sick leave was poor mental health, followed by other conditions (e.g., surgery, cardiopulmonary, abdominal) and musculoskeletal problems. Irrespective of condition, most workers reported at least moderate depression and anxiety at baseline (Table 4). Retention met or approached green feasibility thresholds (workers, 70.5%; managers, 69.2%).

### 3.2. Intervention Fidelity, Acceptability, and Usability

In Phase 1, website analytics were available for 57/91 leaders/HR professionals (Supplementary File S4); 28/91 (30.8%) accessed all content and 18/23 (78%) interviewed reported full engagement. Among those who used the materials and completed follow-up, 31/34 (91.2%) found the training engaging and would recommend it. Participants reported improved understanding of mental health at work (83%) and sickness absence/RTW support (about half), with high baseline scores potentially limiting improvements in readiness to change and communication. Most planned to embed the toolkits into policy and practice (75–83%). Qualitative analyses found that participants liked the balance of video and text in the webinars and the clarity, relevance, and ease of use of the toolkits.

In Phases 2–3, regarding worker fidelity, website analytics showed that 10/43 workers (23.3%) accessed steps 1–5 during Phase 2, 14/43 (32.6%) accessed steps 2–5, and 13/43 (30.2%) accessed step 6 (Supplementary File S5a). Eight workers used printed toolkits, suggesting true engagement exceeded online metrics. In interviews, 26/27 (96.3%) reported completing at least one step in Phase 2 and 14/18 (77.8%) completed step 6 at Phase 3. Among managers, 4/20 (20.0%) accessed steps 1–5 online during Phase 2 and 6/20 (30.0%) accessed step 6 (Supplementary File S5b); some used hard copies or became ineligible because workers moved teams. In interviews, 10/12 (83.3%) managers reported completing at least one step, though none completed all steps.

The findings integrate content, template, and inductive thematic analyses. Acceptability was high: all workers and 80% of managers who used the toolkits rated them positively. Workers frequently described the toolkits as clear, relevant, and supportive during a period often characterized by emotional strain and uncertainty. “I couldn’t even think about work, but [the toolkit] helped me to think about it and not be frightened of going back” (Organization 2). Likert ratings indicated that most workers and managers found checklists and exercises as “useful” or “very useful” (Supplementary Files S6a and S6b) and the content appropriate to the realities of long-term sickness absence and RTW. Workers found the step-by-step format to be clear and applicable across distinct roles and health conditions and valued having supportive content at a challenging time when HR or healthcare input felt limited. As one participant noted, “It’s made [my return] more positive… made me aware of what I can expect or what should be made available for me, and the importance of making contact with colleagues and speaking to your line manager” (Organization 1). However, a few workers found the volume of information difficult to process early in their absence. Managers valued the structure and clarity the toolkit offered, noting that it aligned with expected RTW conversations, supporting “logical” and “methodical” conversations, and was “one of the easier things I’ve had to use.” (Organization 1). Some managers with heavier workloads found the length challenging and suggested more short videos to support engagement. Usability issues were minor and related to accessing material on mobile devices or work computers with security system restrictions. Several participants found printed copies easier to use.

### 3.3. Coaching Engagement and Contribution

Among 43 intervention workers, 25 (58%) attended at least one session and 18 (42%) completed all three. Participants rated the coaching useful (Supplementary File S7). Qualitative findings indicated that coaching was viewed as the mechanism that “glued together” the toolkit by bridging reflective exercises with actionable RTW planning. Participants found that the coaching sessions strengthened motivation, offered a confidential space for emotional containment, and supported the structure and pacing of decisions during a vulnerable period. As one participant put it, “[The coach] was my structure through my whole absence” (Worker, Organization 1).

Workers with high anxiety or cognitive load particularly valued coaching to organize tasks, rehearse conversations, and build confidence to request adjustments. Monthly sessions were seen as appropriate, though some suggested aligning coaching more tightly with toolkit steps.

### 3.4. Behaviour Changes and Adoption

Across interviews and coaching, 23/29 (79%) workers reported specific behaviour changes linked to toolkit use. Workers described improved communication with managers, being more proactive in seeking support, engaging in more structured goal-setting and wellbeing activities, journalling to externalize worries, using readiness-to-return scales to avoid premature RTW, and implementing workplace adjustments or job-crafting strategies such as taking breaks and reducing distractions. One worker described writing exercises as a way of “offloading… it reinforces what I’m thinking about.” (organization 2). A dominant theme for workers was that the toolkit helped them take ownership of their RTW journey. Participants described using the toolkits to monitor progress, as one participant described, “check [things] off, because it’s quite easy, when you’re ill, to forget about these things, or not consider them” (Worker, Organization 1), prepare for work participation, explore workplace adjustments and develop confidence in communicating needs to managers and colleagues. Workers also described improvements in wellbeing through increased self-compassion and a greater understanding of the relationship between their health and work participation.

Managers reported different but complementary changes. These included more frequent well-being check-ins, greater confidence in managing sickness absences and RTW and a more structured and compassionate approach to conversations with staff. One manager explained: “I’d say that I’m quite an empathetic manager myself, but there were things that surprised me, where I’d think ooh, I never thought of asking about that…” (Organization 3). Managers also described using the toolkits to provide more consistent support “right from them going off to returning and the in-between” (Manager, Organization 2), resulting in stronger manager–worker relationships and increased confidence when supporting future cases of long-term sickness absence.

### 3.5. Context–Mechanism–Outcome Patterns

Qualitative findings suggested that intervention engagement and outcomes were contingent upon interactions between contextual factors and intervention mechanisms (Table 5). Overall, workers were more likely to engage with the intervention when they had supportive managers, manageable workloads, and opportunities to implement workplace adjustments or job-crafting strategies. Engagement was also facilitated when workers and managers had organizational permission and managerial support to use intervention resources during work hours and apply learning within routine RTW activities. Several workers described using the intervention to evaluate whether they were genuinely ready to return rather than returning prematurely because of guilt or pressure, suggesting potential benefits for sustaining work participation after return. As one participant explained: “It does make you think about… am I ready, am I not ready, because sometimes I can be one for going back to work too soon, feel guilty for being off…” (Organization 1).

Participants experiencing long-term sick leave for the first time particularly valued the structured guidance provided by the intervention, which reduced uncertainty about what to expect during sickness absence and RTW. One participant described how the communication resources helped them navigate discussions with their manager: “I had no idea really of how to discuss these things with a manager… and then going through the checklist helped focus my mind on communicating with the boss.” (Organization 1). Workers with previous experience of long-term sickness absence often used selected elements of the toolkit to reinforce or extend existing self-management strategies. Participants with physical as well as mental health conditions also reported benefits from the intervention. Some workers with physical health conditions described becoming more aware of the impact that sickness absence had on their psychological wellbeing and recognised the need to actively support their mental health during recovery.

Several mechanisms underpinned participants’ experiences of the intervention. Among workers, these included improved confidence, communication, self-management, awareness of workplace adjustments, and readiness for RTW. Positive outcomes were most reported when workers had sufficient opportunity to implement new strategies and discuss potential adjustments with managers, as one worker discussed: “It’s a really positive, a proactive rather than a reactive tool. It comes across as being a supportive document, if the line manager is involved” (Worker, organization 1). In contrast, high job demands often limited ongoing engagement with the intervention following RTW, particularly with components focused on maintaining wellbeing and job crafting. As one participant explained: “Work wise, patient wise, problems wise it felt like there was an avalanche of work and it wasn’t worth me putting my umbrella up.” (Worker, Organization 1). Conversely, workers whose roles combined manageable demands with sufficient autonomy were better able to continue using toolkit resources and implement changes following RTW.

Among managers, engagement appeared greatest among those who were newer to managing sickness absence or less confident in supporting RTW. These participants frequently described the toolkit as providing practical guidance, reassurance, and greater confidence and capability in managing complex situations, as summed up by one manager: “It will help me in the future because it’s being clear… it’s always good to be able to have something to make sure sickness policy is implemented. But also, it’s done in a way that’s supportive for the staff.” (Organization 1). More experienced managers reported using the materials to validate, reinforce, and refine existing practice rather than acquire entirely new knowledge. Some managers also reported sharing elements of the toolkit with wider teams, suggesting that supportive team cultures may facilitate wider dissemination of intervention principles.

Manager engagement was also influenced by organizational conditions, with those reporting greater autonomy, higher-level support, and time available for well-being-related activities being more likely to engage with toolkit content and apply its recommendations.

Taken together, these findings suggest that intervention outcomes were influenced by interactions between contextual factors and mechanisms operating at the worker, manager, and organizational levels. The findings were used to refine the a priori CMO configurations specified in the published programme theory and trial protocol, resulting in the refined programme theory presented in Table 5.

### 3.6. Organizational Context and Climate-Level Change

Feasibility and engagement were shaped by substantial organizational pressures. Monthly interviews with HR contacts described recruitment freezes, industrial action, staff shortages, and organizational restructuring, all of which limited organizational capacity to support study recruitment and intervention implementation. These pressures were particularly evident in larger public-sector organizations experiencing workforce and service delivery challenges.

HR contacts also identified several organizational barriers to implementation. These included limitations in sickness absence recording systems, difficulties identifying eligible participants, variation in manager involvement in absence management and competing organizational priorities. Although sickness absence policies were broadly similar across participating organizations, differences existed in how consistently policies were implemented, the quality of absence data systems, and the extent to which responsibility for RTW support was devolved to line managers.

Despite these constraints, several intervention organizations reported organizational-level changes associated with participation in the study. Examples included revisions to attendance-management policies, incorporation of toolkit principles into manager development and sickness absence training programmes, and improvements to sickness absence monitoring and reporting procedures. While attribution should be interpreted cautiously, these findings suggest early organizational adoption and integration of intervention principles beyond individual participant use.

Interviews with HR contacts further suggested variability in organizational readiness for implementation. Organizations already undertaking wider initiatives to improve employee health and wellbeing appeared more receptive to integrating intervention principles within existing systems and practices, whereas organizations managing major operational pressures often prioritized immediate workforce challenges over implementation activities.

Organization-wide surveys conducted at baseline and at 18 months (*n* = 871 responding at both points; mean age 44.9, 74% women, 97% White), provided evidence of change at the climate level: mental health stigma decreased in intervention sites relative to controls (mean difference = −0.27, 95% CI −0.49 to −0.04; *p*= 0.02), while perceived leadership support declined in intervention sites (mean difference = −0.21, 95% CI −0.39 to −0.03, *p* = 0.030), likely reflecting organizational restructuring and industrial action reported during the study period. No differences were observed for wellbeing, burnout or organizational communication about mental health resources. Study awareness increased across both trial arms during follow-up.

### 3.7. Secondary (Exploratory) Outcomes

Exploratory outcome analyses were undertaken to assess the suitability of candidate outcome measures and estimate parameters for a future efficacy trial (Supplementary File S8). Outcome data relating to RTW, sustained work participation, and psychological wellbeing were successfully collected across follow-up time points. Time-to-RTW estimates, RTW rates, and sustained work participation rates varied between trial arms, although the study was not designed or powered to evaluate intervention effectiveness.

Economic data was limited: mean organizational cost to implement IGLOo was £9068 over 24 months; the mean per-user cost was £30 for workers and £64 for managers, with toolkit access provided free of charge.

### 3.8. Stop–Go Decision for Main Trial

Organizational willingness met the amber criteria (41.7–42%). Phase 1 recruitment and retention met amber thresholds, while worker and manager recruitment in Phases 2–3 was rated as red (6.2% and 4.2%, respectively). Retention to study-end met the green criteria (workers 71%, managers 69%). Intervention engagement and completion were rated as amber across phases, while acceptability and usability were rated as green (>90% positive ratings among those interviewed). Reported behaviour changes (e.g., managers who are more supportive of workers after their return) exceeded the green benchmarks. Overall, the findings suggest that progression to a future effectiveness trial would require substantial modifications to recruitment pathways, organizational engagement, and implementation support. Although the overall worker recruitment target was achieved across trial arms, recruitment efficiency was substantially lower than anticipated, indicating that much larger pools of eligible participants would be required for a future efficacy trial.

## 4. Discussion

This study examined the feasibility of delivering and evaluating the IGLOo intervention within large UK organizations. The findings indicate that the intervention was acceptable to participants who engaged with it, retention was achievable, outcome data could be collected over 12 months, and implementation mechanisms could be evaluated using a mixed-methods approach. The principal feasibility challenge related to recruitment of workers and managers, highlighting important considerations for future design and implementation.

The most important finding from this feasibility study concerns recruitment. Although the study ultimately recruited a number of workers close to the planned target in the intervention arm and exceeded the overall worker recruitment target across trial arms, recruitment efficiency was substantially lower than anticipated. Organizational recruitment met the predefined amber progression criteria, but worker and manager recruitment rates fell within the red criteria because only a small proportion of invited participants enrolled in the study. As a result, recruitment required considerable organizational effort and access to very large pools of eligible staff. The process evaluation suggests that these challenges arose from a combination of organizational, procedural, and participant-level factors. Organizational restructuring, industrial action, staffing shortages, competing operational priorities, and limitations in sickness absence recording systems all affected recruitment and engagement. In addition, some workers were too unwell to engage at the point of invitation, while managers frequently reported having insufficient time to participate. These findings illustrate the complexities of conducting RTW research in real-world organizational settings and suggest that implementation strategy may be as important as intervention content in determining success. For a future efficacy trial, recruitment pathways will need to become substantially more efficient, requiring stronger organizational commitment, improved identification of eligible workers, and greater integration of intervention activities into existing organizational processes.

Despite these recruitment challenges, the intervention was well-received by workers, managers, and senior leaders who engaged in it. Acceptability and usability consistently met or exceeded predefined progression criteria, while retention rates approached or met target thresholds. Participants described the intervention as relevant, practical, and supportive during a period of considerable uncertainty. Coaching emerged as a particularly valued component, helping workers interpret and apply toolkit content, maintain motivation, and develop plans for returning to work. These findings suggest that the intervention content itself was acceptable and feasible to deliver, even within organizations experiencing considerable operational pressures.

Beyond feasibility outcomes, the study generated preliminary insights into how a multi-level RTW intervention may operate within organizational settings. The findings provide an opportunity to refine the existing IGLOo programme theory using evidence from implementation in real-world organizational settings. The process evaluation identified contextual influences and potential mechanisms operating across individual, managerial, and organizational levels, allowing refinement of the a priori context–mechanisms–outcome configurations specified in the trial protocol [28]. Among workers, these mechanisms included enhanced self-management, confidence to communicate needs, readiness to return, awareness of workplace adjustments, and use of job-crafting strategies. Managers described increased confidence, more structured and compassionate communication, and greater clarity regarding their role in supporting workers during absence and return. At the organizational level, participants highlighted the importance of leadership support, policy integration, and organizational capability to enable implementation. These findings provide preliminary support for the multi-level assumptions underpinning the IGLOo model and identify potential mechanisms that warrant further investigation in a future efficacy trial.

The study also generated important insights into contexts and mechanisms influencing intervention implementation and engagement. Intervention engagement and perceived benefit were not evenly distributed across participants but appeared strongly influenced by contextual conditions. Workers were more likely to engage with and apply intervention content when managers supported toolkit use, work demands were manageable, and there was sufficient opportunity to implement workplace adjustments. Benefits were particularly evident among workers experiencing long-term sick leave for the first time and those requiring greater confidence in navigating the RTW process. Similarly, newer and less experienced managers derived value from the intervention, while more experienced managers often used the materials to validate and refine existing practice. These findings provide valuable information for future intervention targeting and implementation planning.

A further contribution of this study concerns the potential role of organizational conditions in RTW intervention implementation. While participants generally viewed the intervention positively, engagement appeared to be influenced by factors such as leadership support, workload pressures, managerial autonomy and the availability of time to use intervention resources. These findings suggest that organizational readiness may be an important contextual factor influencing implementation, although this requires further investigation.

An important observation for future research concerns the measurement of RTW outcomes. Exploratory analyses suggested different patterns of RTW trajectories across study arms, although any apparent differences between groups should be interpreted cautiously given the small sample size and considerable uncertainty surrounding the effect estimates. Although time-to-initial-return is commonly used as the primary outcome in RTW research, participants frequently emphasized the importance of remaining at work rather than simply returning quickly. Workers described using toolkit exercises to avoid returning prematurely, prepare more effectively for workplace challenges, and negotiate sustainable work arrangements. Taken together, these observations raise the possibility that sustainable work participation may represent a conceptually distinct outcome from rapid RTW and may involve different underlying mechanisms. This remains an important hypothesis arising from the present study. The study therefore supports the inclusion of both timing and sustainability outcomes in future evaluations. Given the feasibility design and imprecision of effect estimates, these observations should be interpreted as hypothesis-generating rather than evidence of intervention effectiveness.

From a public-health perspective, these findings have implications for efforts to address the growing burden of long-term sickness absence and economic inactivity. Current UK policy initiatives such as Get Britain Working [25] and the Keep Britain Working Review [26] increasingly emphasize sustained employability, early intervention, and stronger employer capability in managing health-related work absence. The present study highlights the potential importance of manager capability, structured communication, and organizational support in facilitating work participation among individuals with health conditions. It also illustrates the practical challenges of implementing workplace interventions during periods of organizational instability and workforce pressure. These implementation lessons may be as important as the intervention itself for informing future policy and practice.

### 4.1. Implications for a Future Efficacy Trial

This feasibility study identified several modifications that would be required before progression to a future efficacy trial. The most significant challenge concerned recruitment. Although the target number of workers was ultimately achieved, this required access to a substantially larger population of potentially eligible workers than anticipated, indicating that recruitment pathways need to be considerably more efficient.

A longer set-up and engagement period prior to trial commencement may be necessary to support successful recruitment. This would allow greater integration of study procedures within existing absence-management, occupational health, and human resources processes, and provide sufficient time to establish local implementation procedures. Embedding recruitment within routine organisational practice may reduce the operational burden on participating organisations and improve the identification of eligible workers.

Findings also suggest the importance of increasing organisational awareness of the intervention before recruitment begins. In the present study, some workers received study invitations with little prior knowledge of the intervention. Future trials should consider organisation-wide communication strategies, including manager briefings, staff communications, and awareness campaigns, so that employees are familiar with the intervention and its purpose before potentially receiving an invitation during a period of sickness absence.

Organisational engagement strategies should also be strengthened. Future studies may benefit from identifying senior organisational champions at an earlier stage, assessing organisational readiness for participation, and securing commitments regarding protected time for intervention use and implementation activities. These actions may support both intervention uptake and implementation fidelity.

Implementation procedures may also require further refinement. Greater integration of the toolkits within routine RTW processes, enhanced support for managers experiencing competing workload demands, and continued use of coaching support may improve engagement and facilitate application of intervention learning in practice.

Finally, the findings were derived largely from large public-sector organisations, many of which were experiencing considerable operational pressures during the study period. A future efficacy trial should therefore consider implementation across a wider range of settings to assess transferability and scalability. This could include small and medium-sized enterprises (SMEs), healthcare services, and intermediary organisations, where organisational structures, support systems, and RTW processes may differ substantially. Exploring implementation across diverse organisational contexts would provide a stronger understanding of the adaptability of the intervention and the contextual factors influencing delivery and engagement.

Taken together, these findings suggest that progression to a future efficacy trial is feasible only with modifications to recruitment pathways, organisational engagement strategies and implementation procedures.

### 4.2. Strengths and Limitations

This study has several strengths, including its cluster-randomised design, theory-informed intervention, high retention rates, comprehensive process evaluation and inclusion of multiple stakeholder groups. The mixed-methods approach enabled examination not only of feasibility outcomes but also of implementation processes, contextual influences, and refinement of a priori programme theory. The study also has limitations. Recruitment of workers and managers was substantially lower than intended, limiting confidence in estimates of outcome parameters. The sample was predominantly White and drawn largely from large public-sector organizations, potentially limiting transferability. Furthermore, the intervention was implemented during a period characterized by substantial organizational pressures including restructuring, industrial action, and workforce shortages, which may have reduced engagement with intervention components.

Most participating organizations were drawn from the public sector, including NHS organizations. Private-sector organizations may have different organizational structures, resources, and incentives for RTW support, which may influence both implementation and outcomes.

The realist-informed process evaluation was exploratory and designed to refine an existing programme theory rather than formally test causal relationships between contexts, mechanisms, and outcomes.

Overall, the findings indicate that although the intervention itself was acceptable and showed evidence of meaningful engagement among participants who used it, substantial recruitment and implementation challenges remain. Importantly, while the study ultimately achieved its overall worker recruitment target, this required access to very large numbers of potentially eligible workers and resulted in recruitment rates that fell within predefined red progression criteria. Progression to a future efficacy trial would therefore require major modifications to recruitment pathways, organizational engagement strategies, and implementation support. In its current form, a large-scale trial within similar organizational settings is unlikely to be feasible without these adaptations.

## 5. Conclusions

In summary, this feasibility study demonstrated that delivery and evaluation of the IGLOo intervention is achievable within large organizations, although substantial recruitment challenges need to be addressed before progression to a future efficacy trial. The mixed-methods evaluation identified acceptable intervention components and generated preliminary insights into contextual influences and potential implementation mechanisms that may warrant further evaluation in an efficacy trial. The study also demonstrated the feasibility of collecting outcomes relating to both RTW and longer-term work participation, providing valuable information for the design of a future effectiveness trial. Importantly, although the intervention was acceptable and enabled refinement of the IGLOo programme theory within real-world organizational settings, progression to a future efficacy trial should be considered conditional on substantial improvements to recruitment pathways, organizational engagement, and implementation support.

## Figures and Tables

**Table 1:** Stop–go feasibility criteria and the traffic light * grading assessment.

Outcome	Traffic Light Threshold	Result	Decision
Recruitment			
Organization	Green > 50%; Amber 30–50%; Red < 30%	42%	Amber
Worker	G ≥ 50%; A 30–49%; R < 30%	6.2%	Red
Manager	G ≥ 50%; A 30–49%; R < 30%	4.2%	Red
Retention			
Worker	G ≥ 70%; A 40–69%; R < 40%	70.5%	Green
Manager	G ≥ 70%; A 40–69%; R < 40%	69.2%	Amber
Phase 1 of intervention			
Usability	G ≥ 75%; A 50–74%; R < 50%	91.2%	Green
Completion	G ≥ 50%; A 25–49%; R < 25%	30.8%	Amber
Phase 2 of intervention workers			
Completion	G ≥ 50%; A 25–49%; R < 25%	32.6%	Amber
Acceptability	G ≥ 80%; A 50–79%; R < 50%	41.9%	Amber
Usability	G ≥ 75%; A 50–74%; R < 50%	76% (average)	Green
Coaching attendance (×3)	G ≥ 75%; A 50–74%; R < 50%	100%	Green
Phase 2 of intervention managers			
Completion	G ≥ 50%; A 25–49%; R < 25%	30.0%	Amber
Acceptability	G ≥ 80%; A 50–79%; R < 50%	90.0%	Green
Usability	G ≥ 75%; A 50–74%; R < 50%	91.7% (average)	Green
Phase 3 workers			
Completion	G ≥ 50%; A 25–49%; R < 25%	32.6%	Amber
Behaviour Change	G ≥ 25%; A 10–24%; R < 10%.	79.3%	Green
Phase 3 managers			
Completion	G ≥ 50%; A 25–49%; R < 25%	30.0%	Amber
Behaviour change	G ≥ 25%; A 10–24%; R < 10%	75%	Green

* Green (G) light: main trial to go ahead; amber (A): main trial with adjustments; red (R): no main trial. Completion assessed by web analytics does not include those who may have used a hard copy either by downloading the toolkit or by requesting a hard copy. Usability, acceptability, and behaviour change are based only on those who took part in the interviews.

**Table 2:** Summary of invites and consenting participants for each organization.

Trial Arm	Organization	Size	Invites Sent	Workers Consented	Managers Consented
Intervention	1 [NHS]	~3500	437	27 (6%)	13 (3%)
	2 [NHS]	~5640	287 *	12 (4%)	6 (2%)
	3 [Education]	~2100	33	2 (6%)	1 (3%)
	4 [Transport]	~2006	Unknown	2	0
Control	5 [Law Enforcement]	3337	593	44 (7%)	36 (6%)
	6 [Emergency Services]	~6000	206	7 (3%)	12 (6%)
	7 [Local Authority]	2135	234	8 (3%)	7 (3%)
	8 [Emergency Services]	~961	96	5 (5%)	4 (4%)
	9 [NHS]	6766	67	5 (7%)	4 (6%)

* Estimated figure provided by the organization.

**Table 3:** Demographic data from Phase 1 and Phase 2/3 participants.

	Senior Leaders/HR (Phase 1)	Workers (Phase 2&3)		Managers (Phase 2&3)	
	Intervention Only *n* = 91	Intervention *n* = 43	Control *n* = 69	Intervention *n* = 20	Control *n* = 63
**Gender**					
Male	23 (25%)	9 (21%)	24 (35%)	0 (0%)	26 (41%)
Female	67 (74%)	34 (79%)	45 (65%)	19 (95%)	37 (59%)
Prefer not to say	1 (1%)	0 (0%)	0 (0%)	1 (5%)	0 (0%)
**Ethnicity**					
White	83 (91%)	41 (95%)	63 (91%)	17 (85%)	61 (97%)
Asian	5 (5%)	0 (0%)	2 (3%)	0 (0%)	2 (3%)
Black	0 (0%)	1 (2%)	1 (1%)	0 (0%)	0 (0%)
Mixed	1 (1%)	1 (2%)	1 (1%)	0 (0%)	0 (0%)
Prefer not to say	2 (2%)	0 (0%)	2 (3%)	3 (15%)	0 (0%)
Mean age years (age range)	45 (25–63)	50 (28–67)	44 (21–66)	44 (28–57)	45 (26–62)
**Highest level of education**					
Undergraduate degree or above	64 (70%)	20 (47%)	27 (39%)	9 (45%)	40 (63%)
Below degree level	27 (30%)	23 (53%)	42 (61%)	10 (50%)	23 (37%)
Prefer not to say	0 (0%)	0 (0%)	0 (0%)	1 (5%)	0 (0%)
Mean years in organization [SD]	10.7 [10.2]	12.3 [10.7]	11.5 [9.2]	16.8 [10.1]	15.1 [13.8]
Health conditions *					
Mental health	-	25 (58%)	28 (41%)	11 (55%)	28 (44%)
Musculoskeletal	-	7 (16%)	18 (26%)	4 (20%)	16 (25%)
Other (e.g., heart, lung, surgery)	-	11 (26%)	23 (33%)	5 (25%)	19 (30%)

* For managers, health conditions are for those workers they were managing on sick leave. Health condition categories were not mutually exclusive; participants may have reported more than one condition.

**Table 4:** Mental health scores for control and intervention worker participants.

	Intervention	Control
PHQ9 mean score (SD)		
Mental health	15.6 (6.9)	14.3 (5.3)
Musculoskeletal	9.1 (4.6)	10.0 (6.0)
Other (e.g., heart, lung, surgery)	9.7 (7.7)	9.5 (6.4)
Score of ≥10 on PHQ9 **		
Mental health	24 (56%)	23 (34%)
Musculoskeletal	4 (9%)	11 (16%)
Other (e.g., heart, lung, surgery)	5 (12%)	10 (15%)
GAD-7 mean score (SD)		
Mental health	12.7 (6.1)	12.9 (5.3)
Musculoskeletal	6.3 (4.3)	7.0 (6.8)
Other (e.g., heart, lung, surgery)	7.8 (5.9)	6.1 (5.7)
Score of ≥8 on GAD 7 ^+^		
Mental health	20 (47%)	23 (33%)
Musculoskeletal	4 (9%)	5 (7%)
Other (e.g., heart, lung, surgery)	5(12%)	8 (12%)

** Moderate to severe depression. ^+^ Moderate to severe anxiety.

**Table 5:** Contextual factors, mechanisms, and outcomes facilitating intervention and IGLOo theory refinement.

IGLOo Level	Context	Mechanism	Psychosocial Theory	Outcome	Data
Individual	Workers’ prior knowledge or experience of mental health and RTW.	Used selected toolkit exercises and checklists to extend existing RTW knowledge and skills.	Conservation of Resources	Proximal: Reinforced existing knowledge. Distal: Maintained use of helpful self-management strategies.	Interviews
Individual	Workers absent with mental or physical health conditions.	Increased awareness of the relationship between health, sickness absence and work participation.	Conservation of Resources	Proximal: Greater awareness of mental health during sickness absence. Distal: Adoption of strategies supporting health and recovery.	Interviews
Individual	Manager support for toolkit use.	Had permission to engage with toolkit activities during work hours. Applied toolkit guidance to RTW planning, work adjustments, and job crafting.	Job Demands-Resources	Proximal: Continued toolkit use after RTW. Proximal: Implementation of work and wellbeing adjustments.	Interviews
Individual	Manageable demands and sufficient autonomy.	Had flexibility to engage with toolkits and coaching. Applied learning through, e.g., job crafting and work redesign.	Job Demands-Resources	Proximal: Continued engagement with intervention resources after RTW. Distal: Increased use of job-crafting strategies to support wellbeing.	Interviews
Leader	Less experienced managers.	Used toolkit guidance to develop absence-management skills and RTW support skills.	Conservation of Resources, Social Cognitive	Proximal: Increased confidence and capability in RTW conversations.	Interviews
Leader	Experienced managers seeking improvement.	Used toolkit content to validate and refine existing RTW management practices.	Conservation of Resources, Social Cognitive	Proximal: Reinforcement of effective management behaviours. Distal: Increased confidence in supporting future RTW cases.	Interviews and rating scales
Leader	Organizational support for toolkit use.	Had permission and organisational support to engage with toolkit activities during work hours.	Job Demands-Resources	Proximal: Greater use of structured RTW support practices.	Interviews
Leader	Manager autonomy.	Had flexibility to prioritise and integrate toolkit activities within routine work.	Job Demands-Resources	Proximal: Greater engagement with toolkit resources and wellbeing-supportive practices.	Interviews
Leader and Group	Positive team culture and manager commitment to mental health.	Toolkit messages reinforced manager values and aligned with team norms around wellbeing.	Social Cognitive	Proximal: Sharing of mental health knowledge and supportive practices within teams. Distal: Greater inclusion, support and workplace belonging.	Interviews

## Data Availability

The datasets generated during the study will be available from the corresponding author on reasonable request.

## Supplementary Materials

The following supporting information can be downloaded at: https://www.mdpi.com/article/10.3390/ijerph23081030/s1, Supplementary File S1: IGLOo Model for sustainable return to work, Supplementary File S2: List of Protocol Amendments, Supplementary File S3: Summary of each organisation’s characteristics, Supplementary File S4: Phase 1 intervention web page visits by participants, Supplementary File S5a: Website analytics and interview data on Phase 2 and 3 intervention participant workers, Supplementary File S5b: Website analytics and interview data on Phase 2 and 3 intervention participant managers, Supplementary File S6a: Usefulness of toolkit checklists and exercises for intervention participant workers (Phase 2 *n* = 23; Phase 3 *n* = 16), Supplementary File S6b: Usefulness of toolkit intervention participant managers (Phase 2 *n* = 12; Phase 3 *n* = 6), Supplementary File S7: Usefulness of coaching for intervention participants workers (*n* = 17), Supplementary File 8: Primary and secondary research outcome measures.

## Abbreviations

The following abbreviations are used in this manuscript:

CMO	Context–mechanism–outcome
IGLOo	Individual, Group, Leader, Organization, Outside-context
IOM	Implementation Outcomes Model
IPEF	Integrative Process Evaluation Framework
RTW	Return-to-work
