# Supplementary material for: Supporting Sustainable Return to Work in Large Organizations: A Cluster Randomised Feasibility Trial of a Multi-Level Intervention
Source: Int J Environ Res Public Health. 2026 Aug 6;23(8):1030. doi: 10.3390/ijerph23081030 (PMC13513923; doi:10.3390/ijerph23081030)
Supplement: Supplementary file 1 [file ijerph-23-01030-s001.zip › ijerph-4416299-supplementary.pdf]

Supplementary File S1: IGLOo Model for sustainable return to work

**Level of Resource**	**Example of resources**
Individual	Work-related self-efficacy, health promoting and self-management strategies, job crafting
Group	Understanding and support of colleagues throughout the return-to-work journey
Leader	Knowledge, skills and abilities of leaders and line managers to support the return-to-work journey, implement work adjustments and manage the wider team
Organisation	Knowledge and skills of Human Resources and Occupational Health professionals, policies and practices, training and development opportunities, job design
Outside	Healthcare and charity provision, family and friends outside of work

Adapted from Nielsen et al (2018).

Supplementary File S2: List of Protocol Amendments

1	In phase 2, workers invited during the eligibility window (15–42 days of sick leave) were included even if they responded late due to illness. Managers who delayed returning consent forms due to workload were included if invited within the eligibility window
2	One intervention organisation missed the first two months of Phase 2 recruitment due to staffing issues and relied on senior managers outside central HR to send invitations; the effectiveness of this approach is unclear due to missing updates
3	due to HR capacity constraints, some organisations could not provide sick leave data, so only self-reported data were used for consistency.

Supplementary File S3: Summary of each organisation's characteristics

Group	Organisation number	Size	Type	Workers on LTS+ 12 months prior to study start	LTSA+ rate	Job title of organisational contact
Intervention	1	3500*	NHS	588	17%*	Head of HR
	2	5640	NHS	957	17%	HR Business Partner
	3	2100*	Higher Education	56*	3%*	HR Partner
	4	2,006*	Transport	160	8%*	Wellbeing coordinator
Control	5	3337	Police	445	13%	HR Services Manager
	6	6000*	NHS	1,834	31%*	HR Business Partner
	7	2135	Local Authority	187	9%	OH Safety & Wellbeing Manager
	8	961*	Fire & Rescue	105	11%*	Health and Wellbeing Manager
	9	6766	NHS	929	14%	Chief People Officer

*Estimate; +long-term sick leave absence

Supplementary File S4: Phase 1 intervention web page visits by participants

Page name	Participants n=57 (%)	Mean page visits [SD]	Min– Max page visits	Mean minutes per page [SD]	Median minutes [Interquartile range]	Min – Max minutes
Organisation level introduction page	53 (93%)	2.4 [1.4]	1-6	60.2 [394.9]	2.1 [6.4]	0.2-2880.0
Introduction to hub and toolkits	35 (61%)	1.8 [1.0]	1-4	3.4 [7.9]	1.0 [2.3]	0.1-46.0
Foundations: Where are we now?	40 (70%)	1.9 [1.2]	1-5	8.8 [11.1]	7.0 [11.3]	0.02-51.0
RTW policies and practices	38 (67%)	1.8 [1.3]	1-8	51.2 [234.8]	6.0 [17.4]	0.02-1454.0
Engaging stakeholders for a sustainable return to work	38 (67%)	1.7 [1.0]	1-5	5.8 [12.9]	3.5 [5.8]	0.02-10.0
Setting up for success in your organisation	35 (61%)	1.3 [0.6]	1-3	6.4 [11.1]	2.2 [8.8]	0.02-62.0

Supplementary File S5a: Website analytics and interview data on Phase 2 and 3 intervention participant workers

**Page name**	**Participants** (n=43) n (%)	**Mean page visits** [SD]	**Min– Max page visits**	**Mean minutes per page** [SD]	**Median minutes** [Interquartile range]	**Min – Max minutes**	**Participants interviewed at Phase 2** (n=27) n (%)
Employee intro page	33 (77%)	3.7 [3.4]	1-15	28.3 [74.7]	4.8 [18.1]	0.2-328.7	22 (82%)
Step 1: initial sick leave	24 (56%)	1.8 [1.7]	1-8	4.9 [7.5]	0.6 [10.1]	0.2-29.8	14 (52%)
Step 2: during sick leave	22 (51%)	2.1 [1.2]	1-5	39.5 [86.9]	5.4 [35.4]	0.3-342.1	22 (82%)
Step 3: preparing for return to work	26 (61%)	2.2 [1.5]	1-6	6.5 [10.0]	1.8 [9.8]	0.2-43.8	20 (74%)
Step 4: first week back at work	19 (44%)	2.4 [1.7]	1-6	3.3 [3.4]	3.0 [4.9]	0.2-11.6	17 (63%)
Step 5: keeping healthy and productive at work	18 (42%)	2.8 [2.0]	1-9	3.4 [4.0]	1.9 [6.0]	0.2-13.1	18 (67%)
Accessed steps 1-5	10 (23%)				-		
Accessed steps 2-5	14 (33%)				-		
							**Interviewed at Phase 3** (n=18)
Step 6: job crafting	13 (30%)	3.0 [1.8]	1-7	28.5 [46.9]	3.0 [53.5]	0.2-120.0	14 (78%)
Accessed all steps 1-6	8 (19%)				**-**		

Supplementary File S5b: Website analytics and interview data on Phase 2 and 3 intervention participant managers

Page name	Participants (n=20) n (%)	Mean page visits [SD]	Min– Max page visits	Mean minutes per page [SD]	Median minutes [Interquartile range]	Min – Max minutes	Participants interviewed at Phase 2 (n=12) n (%)
Line manager intro page	11 (55%)	2.4 [1.6]	1-6	2.4 [3.0]	2.0 [3.7]	0.2-10.3	7 (58%)
Step 1: initial sick leave	6 (30%)	1.7 [0.8]	1-3	5.5 [7.3]	3.6 [8.7]	0.1-20.0	7 (58%)
Step 2: during sick leave	7 (35%)	1.4 [0.8]	1-3	1.8 [2.3]	1.0 [1.8]	0.1-6.6	9 (75%)
Step 3: preparing for return to work	9 (45%)	1.2 [0.7]	1-3	5.3 [5.1]	4.4 [7.8]	0.3-15.0	9 (75%)
Step 4: first week back at work	8 (40%)	1.3 [0.5]	1-2	1.7 [2.1]	1.0 [3.0]	0.2-6.0	9 (75%)
Step 5: keeping healthy and productive at work	8 (40%)	1.4 [0.7]	1-3	9.3 [22.5]	0.6 [3.4]	0.2-60.3	6 (50%)
Accessed all steps 1-5	4 (20%)				-		
Accessed all steps 2-5	6 (30%)				-		
							**Interviewed at Phase 3** (n=6)
Step 6: job crafting	6 (30%)	1.2 [0.4]	1-2	1.8 [3.1]	0.8 [2.7]	0.2-8.0	1 (17%)
Accessed all steps 1-6	3 (15%)				-		

Supplementary File S6a: Usefulness of toolkit checklists and exercises for intervention participant workers (Phase 2 n=23; Phase 3 n=16)

Step	Section	No. of participants who gave a rating	Rated 'Useful' or 'Extremely Useful'
Step 1	Main text	12	10
	Step 1: Action Checklist	9	7
	Step 1: Communication Checklist	8	7
Step 2	Main text	19	16
	Step 2: Action Checklist	9	8
	Step 2: Communication Checklist	8	5
	Step 2: Support Network Exercise	14	13
	Step 2: Action Plan Exercise	16	15
	Step 2: Ways to Wellbeing Exercise	14	13
	Step 2: Diary Exercises	8	8
	Step 2: Thoughts About Work Exercise	11	11
Step 3	Main text	18	14
	Step 3: Action Checklist	11	11
	Step 3: Readiness to Return Scale	11	10
	Step 3: Questions & Concerns Exercise	7	6
	Step 3: Possible Work Adjustments Exercise	12	12
	Step 3: Return to Work Conversation Guide	9	8
Step 4	Main text	14	13
	Step 4: Action Checklist	13	13
Step 5	Main text	18	18
	Step 5: Action Checklist	12	12
Step 6	Main text	12	11
	Step 6: Preparing for job crafting	7	6
	Step 6: Exploring job crafting possibilities:	6	5
	Step 6: Developing a job crafting plan	6	5
	Step 6: Action checklist: Discuss and agree a job crafting plan	1	1

Note: There was some missing data on these items; not everyone who said they had used the Step/Checklist/Exercise provided a rating.

Supplementary File S6b: Usefulness of toolkit intervention participant managers (Phase 2 n=12; Phase 3 n=6)

		No. of participants who gave a rating	Rated 'Useful' or 'Extremely Useful'
	Introductory information	9	8*
	Introductory video	7	7
	Return to work conversation guide	3	3
Step 1	Initial sick leave information	8	8
	Action checklist: initial Steps	4	4
	Communications: making first contact	2	2
Step 2	During sick leave: information on what to do	10	9
	Action check list: during sick leave	5	5
	Communication checklist: keeping in touch	4	4
	Communication checklist: coordinated approach	4	4
Step 3	Step 3: Preparing for return-to-work information	9	9
	Step 3 action checklist: preparing for the return	6	6
	Step 3 communication checklist: arranging the meeting	4	4
	Exercise: Possible work adjustments	3	3
	First days and weeks back at work	11	10*
Step 4	Action checklist: supporting the employee	5	5
	Conversation checklist: checking with the employee	3	3
Step 5	Keeping healthy and productive information	7	7
	Action checklist: ongoing review	4	4
Step 6	Job crafting information	1	1

One participant was not asked the question on usefulness

Supplementary File S7: Usefulness of coaching for intervention participants workers *(n=17)*

How useful did you find the coaching in terms of…	No. who answered	Agree (n %)	Totally Agree (n %)
Formulating plans to use the toolkit and stay on track with the toolkit	17	2 (12%)	15 (88%)
Setting goals and identifying actions to support your health and wellbeing	17	-	17(100%)
Finding solutions to return to work barriers	13*	4 (31%)	9 (69%)
Feeling generally supported by the coach	17	-	17 (100%)

*Four participants did not use the coaching in this way because they had already returned to work when they began coaching or used it to focus primarily on supporting their wellbeing.

Supplementary File S8: Primary and secondary research outcome measures

Outcome	Time	Intervention group	N	Control group	N	Effect size (95% CI)[1]
Days to RTW		94 (49,157)	50	60 (34, 86)	92	0.88 (0.49, 1.56)
Days to RTW (employee reported only)		100 (50, 158)	36	59 (39.5, 80.5)	49	0.97 (0.56, 1.68)
Days to RTW (adjusted for covariates)		n/a		n/a		0.91 (0.48, 1.70)
Returned to work?		79%	63	72%	128	1.51 (0.51, 3.26)
Sustainably returned to work?		70%	43	65%	69	1.23 (0.41, 3.58)
Depression	Baseline	22.1 (7.4)	43	20.6 (6.2)	69	n/a
	3 months	17.4 (6.8)	33	16.9 (4.7)	52	-0.11 (-2.69, 2.10)
	6 months	16.4 (6.6)	30	16.3 (5.7)	45	-0.68 (-3.92, 2.05)
	9 months	17.5 (6.8)	30	15.0 (5.2)	47	1.44 (-1.15, 3.91)
	12 months	16.4 (6.0)	28	15.0 (4.8)	46	0.14 (-3.09, 3.15)
Anxiety	Baseline	17.3 (6.3)	43	16.1 (6.6)	69	n/a
	3 months	12.6 (5.9)	33	13.7 (5.6)	52	-1.83 (-4.52, 0.68)
	6 months	13.1 (6.0)	30	11.7 (4.5)	44	1.00 (-1.34, 3.34)
	9 months	14.7 (6.2)	30	11.2 (3.8)	47	2.61 (0.39, 4.61)
	12 months	12.7 (6.0)	28	11.8 (4.8)	46	0.09 (-1.81, 2.69)
Burnout (work-related)	3 months	3.3 (0.9)	27	3.6 (0.8)	38	-0.12 (-0.66, 0.43)
	6 months	3.4 (0.9)	28	3.3 (0.8)	40	0.09 (-0.38, 0.52)
	9 months	3.3 (0.7)	28	3.2 (0.8)	42	0.14 (-0.22, 0.50)
	12 months	3.3 (1.0)	22	3.3 (0.9)	40	0.04 (-0.43, 0.51)
Burnout (non-work-related)	Baseline	3.7 (0.8)	43	3.4 (0.8)	69	n/a
	3 months	3.6 (0.8)	6	3.8 (0.5)	14	-0.34 (-0.89, 0.22)
	6 months	2.7 (0.9)	2	3.5 (0.9)	5	-1.58 (-3.64, 0.46)
	9 months	4.2 (0.2)	2	3.8 (1.1)	5	-0.15
	12 months	3.5 (0.6)	6	3.5 (0.8)	6	-0.03 (-0.82, 0.81)
Quality of life	Baseline	0.6 (0.3)	43	0.6 (0.2)	69	n/a
	3 months	0.2 (2.3)	35	0.5 (1.4)	53	-0.33 (-1.09, 0.45)
	6 months	0.3 (1.8)	31	0.5 (1.5)	45	-0.17 (-1.02, 0.55)
	9 months	0.7 (0.2)	30	0.3 (2.0)	49	0.34 (-0.36, 1.05)

[1] Effect size is adjusted mean difference, except for % returned to work/sustainably returned to work which is an adjusted odds ratio, and Days to RTW, which is an adjusted hazard ratio. Statistics in main columns are mean (SD), except for % returned to work (percentage), and Days to RTW, which is median (IQR)

Outcome	Time	Intervention group	N	Control group	N	Effect size (95% CI)[1]
	12 months	0.7 (0.2)	28	0.7 (0.2)	46	0.02 (-0.10, 0.15)
Confidence in discussing health with manager	Baseline	5.0 (2.0)	43	4.9 (2.1)	69	n/a
	3 months	5.3 (2.1)	33	5.0 (2.0)	51	0.28 (-0.47, 0.96)
	6 months	4.9 (2.0)	30	5.0 (2.0)	44	0.16 (-0.63, 0.86)
	9 months	4.7 (2.1)	29	5.4 (1.8)	47	-0.58 (-1.34, 0.18)
	12 months	5.2 (1.8)	28	4.9 (2.0)	46	0.49 (-0.43, 1.25)
Confidence in discussing health with colleagues	Baseline	3.9 (2.1)	43	4.3 (2.2)	69	n/a
	3 months	4.5 (2.2)	33	4.3 (2.0)	51	0.32 (-0.39, 1.03)
	6 months	4.4 (1.9)	30	4.5 (1.9)	44	0.21 (-0.44, 0.87)
	9 months	4.3 (2.0)	29	4.9 (1.8)	47	-0.49 (-1.16, 0.18)
	12 months	4.4 (1.9)	28	4.5 (2.1)	46	0.02 (-0.74, 0.78)
Confidence in discussing health with friends & family	Baseline	5.0 (1.9)	43	5.7 (1.6)	69	n/a
	3 months	5.1 (1.7)	33	5.5 (1.7)	51	-0.20 (-1.17, 0.68)
	6 months	5.1 (1.8)	30	5.5 (1.5)	44	-0.02 (-0.63, 0.68)
	9 months	5.0 (1.9)	29	5.7 (1.4)	47	-0.06 (-0.69, 0.55)
	12 months	5.4 (1.8)	28	5.7 (1.5)	46	0.04 (-0.65, 0.72)
Satisfaction with frequency of communication on sick leave	Baseline	3.5 (1.1)	43	3.4 (1.3)	69	n/a
	Follow-up	3.9 (1.2)	35	3.7 (1.4)	52	0.24 (-0.27, 0.69)
Satisfaction with support from manager on sick leave	Baseline	3.6 (1.1)	43	3.6 (1.4)	69	n/a
	Follow-up	3.9 (1.2)	35	3.7 (1.3)	52	0.24 (-0.26, 0.66)
Job satisfaction	Baseline	4.6 (1.8)	43	4.5 (1.5)	69	n/a
	3 months	4.5 (1.8)	27	4.5 (1.6)	38	-0.02 (-0.65, 0.61)
	6 months	4.5 (1.4)	28	4.7 (1.7)	40	-0.20 (-0.83, 0.55)
	9 months	4.6 (1.5)	28	5.0 (1.5)	42	-0.39 (-1.04, 0.23)
	12 months	4.4 (1.8)	22	4.7 (1.4)	40	-0.23 (-0.98, 0.53)
Intention to quit	Baseline	2.5 (1.1)	43	2.7 (1.2)	69	n/a
	3 months	2.8 (1.3)	33	2.7 (1.2)	51	0.26 (-0.11, 0.64)
	6 months	2.6 (1.3)	30	2.6 (1.2)	44	-0.01 (-0.47, 0.48)
	9 months	2.7 (1.3)	29	2.5 (1.3)	47	0.28 (-0.24, 0.79)
	12 months	2.6 (1.3)	28	2.5 (1.3)	46	0.15 (-0.39, 0.72)
Readiness to stay	3 months	3.7 (0.7)	27	3.6 (0.6)	38	0.13 (-0.18, 0.48)
	6 months	3.7 (0.7)	29	3.7 (0.6)	40	0.00 (-0.31, 0.34)

Outcome	Time	Intervention group	N	Control group	N	Effect size (95% CI)[1]
	9 months	3.7 (0.8)	28	3.8 (0.6)	43	-0.14 (-0.48, 0.19)
	12 months	3.8 (0.8)	22	3.8 (0.6)	40	0.00 (-0.34, 0.34)
Line manager competency - managing sickness absence	Follow-up	3.9 (1.2)	33	3.6 (1.2)	47	0.23 (-0.31, 0.77)
Line manager competency - managing RTW	Follow-up	3.8 (1.0)	34	3.3 (1.3)	43	0.41 (-0.12, 0.95)
Manager support	3 months	3.4 (0.9)	27	3.5 (0.9)	38	-0.09 (-0.54, 0.39)
	6 months	3.3 (0.9)	28	3.3 (1.0)	40	-0.06 (-0.67, 0.52)
	9 months	3.4 (0.9)	28	3.7 (0.9)	42	-0.31 (-0.79, 0.13)
	12 months	3.3 (1.0)	22	3.5 (0.9)	40	-0.27 (-0.78, 0.20)
Job crafting	3 months	3.0 (0.4)	27	3.1 (0.6)	38	-0.23 (-0.62, 0.16)
	6 months	2.9 (0.5)	28	2.9 (0.4)	40	-0.06 (-0.28, 0.16)
	9 months	2.9 (0.4)	28	3.0 (0.5)	42	-0.12 (-0.34, 0.09)
	12 months	2.9 (0.5)	22	3.0 (0.5)	40	-0.16 (-0.35, 0.14)
Colleague support	3 months	3.6 (1.0)	27	3.9 (1.1)	38	-0.29 (-0.81, 0.23)
	6 months	3.8 (1.0)	28	3.9 (1.2)	40	-0.05 (-0.59, 0.49)
	9 months	3.7 (1.0)	28	4.2 (0.8)	42	-0.51 (-0.97, -0.08)
	12 months	3.5 (1.0)	22	4.0 (0.9)	40	-0.45 (-1.01, 0.04)
Job autonomy	3 months	4.6 (1.3)	27	4.4 (1.5)	38	0.24 (-0.45, 0.94)
	6 months	4.7 (1.3)	28	4.4 (1.4)	40	0.36 (-0.41, 1.03)
	9 months	4.6 (1.3)	28	4.5 (1.7)	42	0.14 (-0.58, 0.87)
	12 months	4.6 (1.5)	22	4.1 (1.7)	40	0.50 (-0.38, 1.34)
Job demands	3 months	2.6 (1.0)	27	3.0 (1.0)	38	-0.38 (-1.02, 0.26)
	6 months	2.5 (0.8)	28	2.7 (0.8)	40	-0.26 (-0.81, 0.28)
	9 months	2.5 (0.7)	28	2.6 (0.8)	42	-0.08 (-0.49, 0.33)
	12 months	2.5 (0.9)	22	2.7 (0.9)	40	-0.13 (-0.60, 0.37)
**Results from managers' surveys**						
Manager - competence in managing sickness absence	Follow-up	5.0 (0.3)	14	4.7 (0.5)	46	0.28 (0.01, 0.55)
Manager - competence in managing RTW	Follow-up	5.0 (0.3)	14	4.4 (0.7)	46	0.52 (0.16, 0.88)
Manager - confidence in managing mental health issues	Baseline	3.5 (0.8)	20	3.9 (0.7)	62	n/a
	3 months	4.2 (0.7)	14	4.0 (0.6)	50	0.33 (0.08, 0.58)
	6 months	4.1 (0.7)	7	4.0 (0.5)	40	0.25 (-0.08, 0.59)
	9 months	4.5 (0.4)	5	4.1 (0.5)	34	0.32 (-0.01, 0.65)

Outcome	Time	Intervention group	N	Control group	N	Effect size (95% CI)[1]
	12 months	4.5 (0.6)	7	4.2 (0.4)	28	0.25 (-0.08, 0.57)
Manager job demands	Baseline	2.8 (1.1)	20	2.8 (0.9)	62	n/a
	12 months	2.5 (1.0)	7	2.8 (0.8)	28	-0.28 (-0.64, 0.08)
Manager autonomy	Baseline	5.4 (1.3)	20	5.2 (1.3)	62	n/a
	12 months	6.0 (1.1)	7	5.3 (1.2)	28	0.58 (-0.97, 2.13)
